# Fingolimod Modulates Dendritic Architecture in a BDNF-Dependent Manner

**DOI:** 10.3390/ijms21093079

**Published:** 2020-04-27

**Authors:** Abhisarika Patnaik, Eleonora Spiombi, Angelisa Frasca, Nicoletta Landsberger, Marta Zagrebelsky, Martin Korte

**Affiliations:** 1 Zoological Institute, Division of Cellular Neurobiology, TU Braunschweig, D-38106 Braunschweig, Germany; a.patnaik@tu-bs.de; 2 Department of Medical Biotechnology and Translational Medicine, University of Milan, 20100 Milan, Italy; eleonora.spiombi@unimi.it (E.S.); Angelisa.frasca@unimi.it (A.F.); nicoletta.landsberger@unimi.it (N.L.); 3 Helmholtz Centre for Infection Research, AG NIND, Inhoffenstr. 7, D-38124 Braunschweig, Germany

**Keywords:** Fingolimod, FTY720, BDNF, primary cultures, dendrites, dendritic spines, Rett syndrome, *Mecp2*, *Cdkl5*

## Abstract

The brain-derived neurotrophic factor (BDNF) plays crucial roles in both the developing and mature brain. Moreover, alterations in BDNF levels are correlated with the cognitive impairment observed in several neurological diseases. Among the different therapeutic strategies developed to improve endogenous BDNF levels is the administration of the BDNF-inducing drug Fingolimod, an agonist of the sphingosine-1-phosphate receptor. Fingolimod treatment was shown to rescue diverse symptoms associated with several neurological conditions (i.e., Alzheimer disease, Rett syndrome). However, the cellular mechanisms through which Fingolimod mediates its BDNF-dependent therapeutic effects remain unclear. We show that Fingolimod regulates the dendritic architecture, dendritic spine density and morphology of healthy mature primary hippocampal neurons. Moreover, the application of Fingolimod upregulates the expression of activity-related proteins c-Fos and pERK1/2 in these cells. Importantly, we show that BDNF release is required for these actions of Fingolimod. As alterations in neuronal structure underlie cognitive impairment, we tested whether Fingolimod application might prevent the abnormalities in neuronal structure typical of two neurodevelopmental disorders, namely Rett syndrome and Cdk5 deficiency disorder. We found a significant rescue in the neurite architecture of developing cortical neurons from *Mecp2* and *Cdkl5* mutant mice. Our study provides insights into understanding the BDNF-dependent therapeutic actions of Fingolimod.

## 1. Introduction

The secreted growth factor brain derived neurotrophic factor (BDNF) belongs to the Neurotrophin family and is produced by neurons in an activity-dependent manner in all major brain areas [1]. It interacts with the tropomyosin receptor kinase B (TrkB) to accomplish its trophic and plasticity-promoting activities. In the developing central nervous system (CNS), BDNF enhances cell survival, promotes morphogenesis and differentiation, and contributes to the formation and maturation of synapses [2]. In the mature CNS, BDNF is essential for activity-dependent synaptic plasticity, regulating synaptic transmission, and maintaining the mature neuronal architecture, including dendritic spine density, size and shape [3,4], as well as for learning and memory processes [5]. The expression of BDNF is tightly regulated and alterations in BDNF levels and function have been suggested to underlie both the cognitive decline during aging processes as well as the pathogenesis of several neurological and psychiatric disorders [6,7,8]. While BDNF has been shown to exhibit potent therapeutic effects, its delivery to the brain remains challenging due to its short half-life and poor diffusion across the blood-brain-barrier. Current therapeutic strategies employ viral-mediated *Bdnf* gene delivery or modulation of TrkB activity to promote BDNF-TrkB signaling. Alternatively, the administration of BDNF-inducing drugs has been shown to rescue different neurological diseases characterized by BDNF dysregulation. Of special interest in this context is the sphingosine-1-phosphate analog Fingolimod, an immunomodulatory oral drug used in relapsing-remitting forms of multiple sclerosis [9]. The pro-drug (Fingolimod) diffuses across the blood–brain barrier into the CNS parenchyma where it is phosphorylated into its active form Fingolimod-P and acts as ligand to a subset of Sphingosine-1-phosphate receptors (S1PR1 and 3–5) expressed, among others, also by different brain cells [10]. Interestingly, Fingolimod has been shown to upregulate BDNF mRNA levels and increase BDNF protein release both in vitro and in vivo in an activity- and MAPK-dependent manner [11]. The functional significance of the Fingolimod-induced BDNF release is marked by its ability to improve the cognitive impairment typical of different neurological diseases, e.g., Alzheimer’s and Huntington’s diseases [12,13]. Furthermore, the rescue of typical symptoms upon Fingolimod treatment was shown to be associated with improved synaptic plasticity and amelioration of dendritic spine loss in the hippocampus of a mouse model for Huntington’s disease [13] and prevented spine loss in the cortex during the acute phase of experimental acute encephalitis [14,15]. 

Rett syndrome (RTT) is an X-linked neurodevelopmental genetic disorder mainly due to loss of the *Mecp2* function resulting at the cellular level in impaired neurite outgrowth and abnormal neuronal architecture [16,17] associated with the reduction of both BDNF mRNA and protein levels [18]. BDNF overexpression, its exogenous application, or TrkB agonistic activation have been shown to restore synaptic plasticity, improve neuronal activity and structural aberrations in *Mecp2* deficient mice, mimicking the human disorder [18,19,20]. Moreover, Fingolimod administration increased BDNF levels in the cortex, hippocampus, and striatum and significantly improved the motor deficits observed in *Mecp2* mutant mice [11]. Cdkl5 deficiency disorder (CDD), an X-linked atypical variant of RTT is also characterized by impairment in neuronal morphology [21,22] and reduced BDNF mRNA levels [22]. Treatment with a specific TrkB agonist was shown to rescue the structural and functional impairments in a Cdkl5 deficient mouse [23] indicating an important role of the BDNF-TrkB signaling in this context. 

While Fingolimod has been established as a promising therapeutic agent for several disease models, whether it is able to modulate the structure of mature, healthy CNS neurons is currently not known. Here, we examine the ability of Fingolimod to modulate dendritic architecture in a BDNF-dependent manner of primary wild type hippocampal neurons. Moreover, in our study, we tested whether Fingolimod application can rescue structural anomalies in cortical neurons of *Mecp2* and *Cdkl5* mutant mice.

## 2. Results

### 2.1. Fingolimod-Phosphate (FTY720-P) Modulates Dendritic Architecture of Mature, Healthy Hippocampal Neurons 

**Figure 1:**
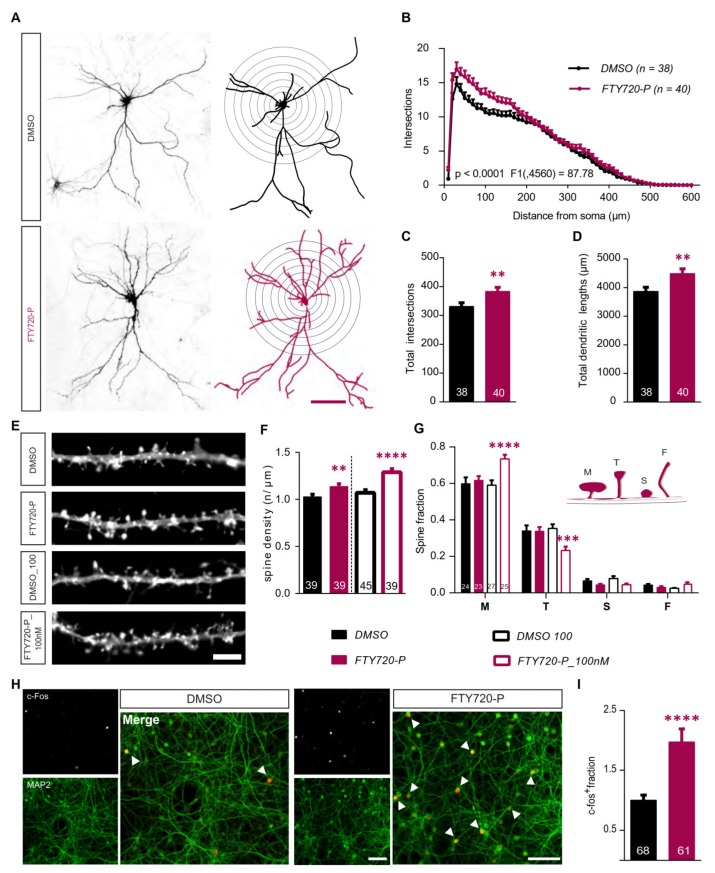
Fingolimod-phosphate (FTY720-P) modulates neuronal morphology in mature hippocampal neurons. FeGFP expressing 21DIV primary hippocampal neurons from healthy wild type BL/6J mice were treated with 10nM FTY720-P for 24h. (**A**) Representative images (left) of feGFP expressing neurons treated with DMSO (above, black) and 10nM FTY720-P (below, magenta) and the relative Neurolucida tracing used to perform the Sholl analysis (right). Scale bar: 100μm. (**B**) Sholl analysis displaying dendritic complexity plotted against distance from the cell soma. *F* value refers to the DMSO v/s FTY720-P comparison. The graphs show (**C**) the total dendritic complexity and (**D**) total lengths of dendrites for the DMSO (black) and FTY720-P (magenta) treated neurons. (**E**) Representative segments of secondary dendrites from feGFP transfected neurons used to analyze dendritic spine density. Scale bar: 5μm (**F**) Graphical representation of the spine density for neurons treated with 10 (magenta solid) or 100nM (magenta open) FTY720-P their respective DMSO controls (10nM black solid; 100nM black open). (**G**) the graph shows the distribution of different dendritic spine types across all treatment groups. The four different types of spines– mushroom (M), thin (T), stubby (S) and filopodia (F) as represented in the schematic, were quantified and represented as a fraction of total spines for neurons treated with FYT720P (10nM magenta solid; 100nM magenta open) or DMSO (10nM black solid; 100nM black open). (**H**) Images of fields of view (FOV) of DMSO (left) and FTY720-P (right) treated primary hippocampal cultures immunostained for c-Fos (inset: above) and MAP2 (inset: below) with their respective merged images (right). The arrows indicate c-Fos expressing MAP2 positive neurons in the merged picture. Scale bar: 100μm. (**I**) Graph comparing the normalized number of c-Fos^+^ cells over total MAP2^+^ neurons between DMSO (black) and FTY720-P (magenta) treated cultures. All plots represent data as mean + SEM. Numbers in the bars indicate total number of neurons or of FOV analyzed, obtained from ≥3 sets of independent experiments. Two-way ANOVA followed by Bonferroni post-hoc test was used in (**B**) and (**G**). For (**C,D**) and (**I**) unpaired Student’s *t*-test and for (**F**), one-way ANOVA with Bonferroni post-hoc was used. Significance is denoted as ** *p* < 0.01, *** *p* < 0.001, **** *p* < 0.0001.

To address a potential role of Fingolimod-phosphate (FTY720-P) in modulating the dendritic architecture of healthy hippocampal neurons, 21 DIV primary hippocampal cultures were treated for 24h with 10 nM FTY720-P or DMSO, as control. The Sholl analysis, performed on feGFP expressing hippocampal neurons, showed a significantly higher dendritic complexity upon FTY720-P treatment when compared to controls (Figure 1A). The increase in dendritic complexity was found to be statistically significant specifically for the portion of the dendritic tree closer to the cell soma (Figure 1A,B; two-way ANOVA *F*_1,4560_ = 87.78, *p* < 0.0001). Accordingly, a significant increase was observed in the total number of dendritic intersections (Figure 1C; two-tailed unpaired t-test: *t* = 2.710, *df* = 6, *p* < 0.01; DMSO: 330.8 ± 13.10 vs, FTY720-P: 383.4 ± 14.20) as well as in the total dendritic lengths of FTY720-P treated neurons compared to controls (Figure 1D; two-tailed unpaired *t*-test: *t* = 2.820, df = 76, *p* < 0.01; DMSO: 3867 ± 142.9 vs. FTY720-P: 4488 ± 166.0). 

Next, to determine if FTY720-P also influences dendritic spines number, dendritic spine density was quantified on secondary dendritic branches of control and FTY720-P treated neurons (Figure 1E). We observed a significant increase (~10%) in the number of dendritic spines per μm in the FTY720-P group (Figure 1E,F; two-tailed unpaired *t*-test: *t* = 2.669, *df* = 76, *p* < 0.01; DMSO: 1.027 ± 0.0283, FTY720-P: 1.137 ± 0.0299). Subsequently, a possible concentration-dependent activity of FTY720-P in regulating dendritic spine density was tested by treating primary hippocampal cultures with a 10-fold higher concentration of FTY720-P (100 nM) or its respective DMSO control. The analysis revealed that, in comparison to the control, the higher drug concentration significantly enhanced spine density by ~20% (Figure 1E,F; two-tailed unpaired t-test: *t* = 4.369, *df* = 82, *p* < 0.0001; DMSO: 1.071 ± 0.0334, FTY720-P_100nM: 1.289 ± 0.0373). Next, a possible concentration-dependent role of FTY720-P was tested in modulating dendritic spine morphology. For this purpose, dendritic spines were structurally classified into four subtypes-mushroom, thin, stubby or filopodia-like (Figure 1G, schematic) based on their length and their head-to-neck ratios [24]. While the neurons treated with lower FTY720-P concentration (10 nM) did not show any difference in their spine-type distribution compared to the relative control neurons, treatment with higher FTY720-P concentration (100 nM) showed a significant increase in the proportion of mushroom-type spines (Figure 1E,G; *p* < 0.0001; DMSO: 0.591 ± 0.027 vs. FTY720-P: 0.735 ± 0.023) associated with a significant decrease in the proportion of thin spines (Figure 1E,G; *p* < 0.001; DMSO: 0.353 ± 0.023 vs. FTY720-P: 0.233 ± 0.021). The fraction of stubby and filopodia-like spines remained unchanged across all treatment groups (Figure 1E,G).

Overall, these observations indicate that, in mature primary hippocampal neurons, a 24-h treatment with FTY720-P results in a mild increase in the dendritic complexity, particularly at the proximal dendrites and in total dendritic length. Moreover, a FTY720-P treatment affects the dendritic spine density and structure in a concentration-dependent manner, with the higher concentration inducing an increase in the proportion of mature dendritic spines. 

### 2.2. Fingolimod-Phosphate induces the Expression of the Activity-Related Immediate Early Gene c-fos

FTY720-P has been shown to positively modulate network activity of cultured cortical neurons by promoting excitatory and suppressing inhibitory synaptic transmission signaling via the S1PR1 [11]. Moreover, treatment with FTY720-P induces the phosphorylation of the activity-related kinases ERK1/2 and CREB in primary cortical neurons [11] and FTY720 has been shown to upregulate the activity-related immediate early gene *c-fos* in cultivated cerebellar neurons [25]. Therefore, we next tested whether FTY720-P can also enhance c-Fos protein expression in hippocampal neurons. Therefore, primary hippocampal cultures treated for 24h with FTY720-P or DMSO were immunostained for c-Fos and the number of c-Fos (c-Fos^+^, Figure 1H, inset above) and MAP2 (MAP2*^+^*, Figure 1H, inset below) immunoreactive neurons was quantified. Both treatment groups displayed a clear nuclear localization of c-Fos in neurons, identified by their immune-positivity for MAP2 (Figure 1H). However, in the control group only few neurons were positive for c-Fos in comparison to those treated with FTY720-P (Figure 1H). Quantification of c-Fos^+^/MAP2^+^ fraction after FTY720-P treatment revealed a significant, almost two-fold higher number in c-Fos^+^ neurons than under control conditions (Figure 1I; two-tailed unpaired *t*-test: *t* = 4.337, *df* = 127, *p* < 0.0001; DMSO: 1.000 ± 0.0856, FTY720-P: 1.975 ± 0.2174). 

This observation shows the ability of a 24-h treatment with FTY720-P to upregulate c-Fos transcription factor in primary hippocampal neurons. 

### 2.3. Fingolimod-Phosphate (FTY720-P) Mediated Effects are BDNF-Dependent

**Figure 2:**
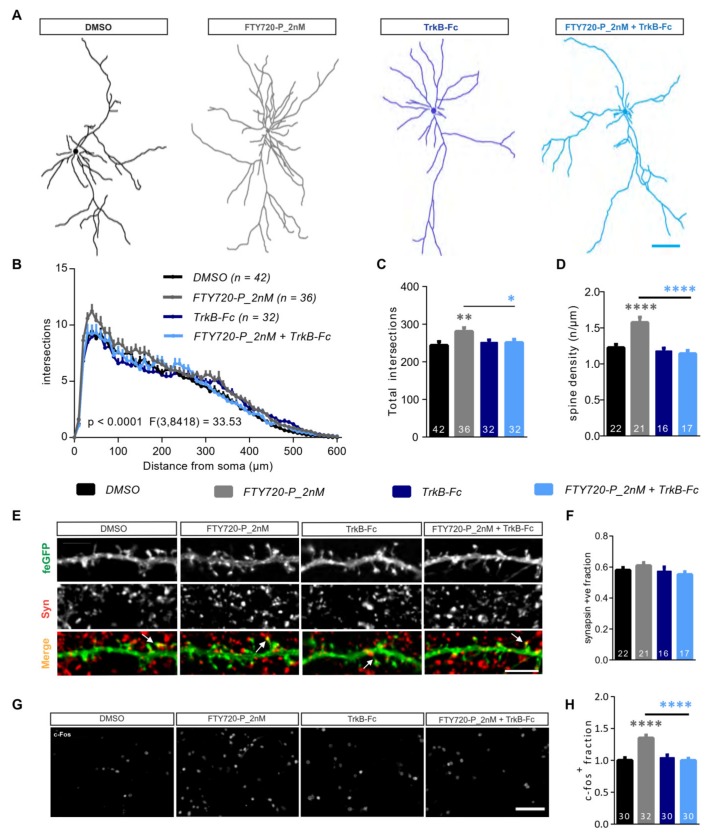
Fingolimod-phosphate (FTY720-P) regulates neuronal architecture in a BDNF-dependent manner. (**A**) Representative Neurolucida tracings, used to perform the Sholl analysis of dendritic complexity for feGFP expressing neurons treated for 24h with: DMSO (black), 2nM FTY720-P (gray), DMSO + TrkBFc (dark blue) and 2nM FTY720-P + TrkBFc (light blue). Scale bar: 100 μm. (**B**) Sholl analysis displayed as number of dendritic intersections against distance from the cell body for DMSO (black), 2nM FTY720-P (gray), DMSO + TrkBFc (dark blue) and 2nM FTY720-P + TrkBFc (light blue) and (**C**) total dendritic complexity of all treatment groups. *F* value in (**B**) refers to comparison between all the 4 treatment groups. (**D**) Dendritic spine densities for DMSO (black), 2nM FTY720-P (gray), DMSO + TrkBFc (dark blue) and 2nM FTY720-P + TrkBFc (light blue) calculated using segments of secondary dendritic branches as shown in (**E**) feGFP panel. The Syn panel displays the corresponding staining of the pre-synaptic marker SynapsinI/II and the merge panel shows the images with overlapping SynapsinI/II puncta (red, pre-synapse) to its matching feGFP dendrite segment (green, post-synapse). The arrows point to coinciding puncta, indicative of mature synapse between the post and pre-synaptic compartments. Scale bar: 5μm. (**F**) The graph compares the fraction of SynapsinI/II positive feGFP labelled spines for DMSO (black), 2nM FTY720-P (gray), DMSO + TrkBFc (dark blue) and 2nM FTY720-P + TrkBFc (light blue). (**G**) Representative fields of view (FOV) of the DMSO, 2nM FTY720-P, DMSO + TrkBFc and 2nM FTY720-P + TrkBFc treated hippocampal cultures stained for c-fo*s*. Scale bar: 100μm. (**H**) Quantification of the proportion of c-Fos expressing neurons represented as normalized fraction for DMSO (black), 2nM FTY720-P (gray), DMSO + TrkBFc (dark blue) and 2nM FTY720-P + TrkBFc (light blue) treated cultures. All graphs represent data as mean + SEM. Numbers in the bars show either total number of neurons or of FOV analyzed, obtained from ≥3 sets of independent experiments. Two-way ANOVA followed by Bonferroni post-hoc test was used in (**B**). For (**C**,**D**,**F**) and (**H**) one-way ANOVA with Bonferroni post-hoc was used. Denotations for significance are * *p* < 0.05, ** *p* < 0.01, **** *p* < 0.0001.

Several studies have reported the ability of FTY720 and/or FTY720-P to positively modulate the expression and the secretion of BDNF both in vitro and in vivo [11,12,15,26]. Deogracias et al. (2012) showed a time-dependent increase in BDNF mRNA and protein levels with a peak after 24h FTY720-P application in primary cortical neurons. Interestingly, the increase in BDNF protein was shown to be inversely correlated with the drug concentration [11]. A low dosage of FTY720 was also reported to influence in vitro viability of neural stem cells [27] and recently, also to rescue memory impairment in a mouse model of Alzheimer’s disease [28]. Thus, we next tested the activity of a 5-fold lower concentration of FTY720-P in modulating the dendritic architecture of hippocampal neurons in primary hippocampal cultures. DIV21 feGFP expressing neurons were treated for 24 h with 2 nM FTY720-P (FTY720-P_2nM) or DMSO and dendritic complexity was compared using the Sholl analysis (Figure 2A). Interestingly, this low FTY720-P concentration also led to significant alterations in the complexity of proximal dendrites (Figure 2A,B; two-way ANOVA *F*_3,8418_ = 33.53, *p* < 0.0001). Indeed, a significantly higher number of intersections were observed, especially between 20 and 50 μm from the cell body (Figure 2A,B) compared to DMSO treated neurons. Likewise, significantly more total intersections were computed for neurons treated with 2 nM (FTY720-P_2nM) than for the neurons treated with DMSO (Figure 2C; one-way ANOVA *F*_3,138_ = 3.469, *p* < 0.05; DMSO: 243.9 ± 9.940, FTY720-P_2nM: 281.0 ± 9.429).

FTY720 has been shown to modulate BDNF levels in a time and concentration-dependent manner. BDNF, which is a critical modulator of structural and functional plasticity in neurons [3], promotes dendritic growth and branching [29,30], and regulates dendritic spine density and morphology in mature neurons [31,32,33]. Therefore, we asked whether an increase in BDNF release is required for the FTY720-P induced changes in the dendritic architecture. To achieve this aim, DIV21 primary hippocampal cultures were treated with a either FTY720-P_2nM or TrkB receptor bodies (TrkB-Fc) or a combination of the two for 24 h. Treatment with TrkB-Fc alone did not alter dendritic morphology compared to the relative DMSO control as measured using Sholl analysis (Figure 2A,B; two-way ANOVA *F*_3,8418_ = 33.53, *p* < 0.0001). However, when applied in combination with TrkB-Fc, a 24-h treatment with FTY720-P_2nM failed to induce the significant modifications in the dendritic architecture observed upon application of FTY720-P_2nM alone (Figure 2A,B). Indeed, also the increase in total intersections upon FTY720-P_2nM treatment was completely abolished through the TrkB-Fc co-treatment and reduced to control levels (Figure 2C; one-way ANOVA *F*_3,138_ = 3.469, *p* < 0.05; DMSO: 243.9 ± 9.940, FTY720-P_2nM: 281.0 ± 9.429, TrkB-Fc: 251.8 ± 6.460, FTY720-P_2nM+TrkB-Fc: 251.0 ± 8.672). Further, a BDNF-dependent effect of FTY720-P_2nM in regulating the number of dendritic spines was analyzed on secondary dendritic branches of primary hippocampal neurons. While a treatment with TrkB-Fc alone resulted only in a mild decrease in dendritic spine density, a 24-h application of FTY720-P_2nM significantly augmented spine density relative to the control treated neurons (Figure 2E-feGFP, D; one-way ANOVA *F*_3,72_ = 13.66, *p* < 0.0001; DMSO: 1.226 ± 0.0449, FTY720-P_2nM: 1.574 ± 0.0736). This increase was completely prevented when the FTY720-P_2nM was co-applied with TrkB-Fc (Figure 2E-feGFP, D; one-way ANOVA *F*_3,72_ = 13.66, *p* < 0.0001; DMSO: 1.226 ± 0.0449, FTY720-P_2nM: 1.574 ± 0.0736, TrkB-Fc: 1.180 ± 0.0388, FTY720-P_2nM+TrkB-Fc: 1.142 ± 0.0451). To probe whether the increase in dendritic spine density corresponds to an increase in the absolute number of functional synapses, the cultures were immunostained for the pre-synaptic marker SynapsinI/II (Figure 2E-Syn) and its co-localization with the dendritic spines of feGFP transfected neurons was compared between the different treatments (Figure 2E-Merge). Across all treatment groups, the proportion of synapsinI/II positive spines was found to be about ~57% with only a slight increase upon FTY720-P_2nM treatment and a slight decrease upon the combined treatment with FTY720_2nM and TrkB-Fc (Figure 2E-Merge, F; one-way ANOVA *F*_3,72_ = 1.398, *p* = 0.2505; DMSO: 0.5800 ± 0.0168, FTY720-P_2nM: 0.6093 ± 0.0194, TrkB-Fc: 0.5740 ± 0.0267, FTY720-P_2nM+TrkB-Fc: 0.5516 ± 0.0183). These differences, however, did not reach significance (Figure 2F). This constant fraction of SynapsinI/II positive, mature synapses suggest that FTY720-P_2nM indeed increases the absolute number of functional synapses (Figure 2E-Merge, F).

In the CNS, *c-fos* is primarily associated with the regulation of neuronal plasticity [34] and has been shown to be activated downstream of BDNF signaling [33,35]. Thus, we next assessed whether the FTY720-P-induced increase in the proportion of c-Fos positive neurons also depends upon the release of BDNF. Indeed, while treatment with 2nM FTY720-P significantly increased the number of c-Fos positive neurons, combining it with TrkB-Fc completely prevented the enhancement in the number of c-Fos expressing neurons (Figure 2G,H; one-way ANOVA *F*_3,118_ = 17.78, *p* < 0.0001; DMSO: 1.000 ± 0.0414, FTY720-P_2nM: 1.350 ± 0.0454, TrkB-Fc: 1.043 ± 0.0404, FTY720-P_2nM+TrkB-Fc: 0.9985 ± 0.0340).

Taken together, these observations demonstrate that low concentrations of FTY720-P efficiently modulate dendritic architecture and neuronal activation in mature, healthy primary hippocampal neurons. Moreover, the TrkB-Fc co-treatment results indicate a crucial role for the release of BDNF in mediating the effects of FTY720-P.

### 2.4. Non-Phosphorylated Fingolimod (FTY720) Recapitulates the Dendritic Modifications Induced by the Phosphorylated (FTY720-P) Form

**Figure 3:**
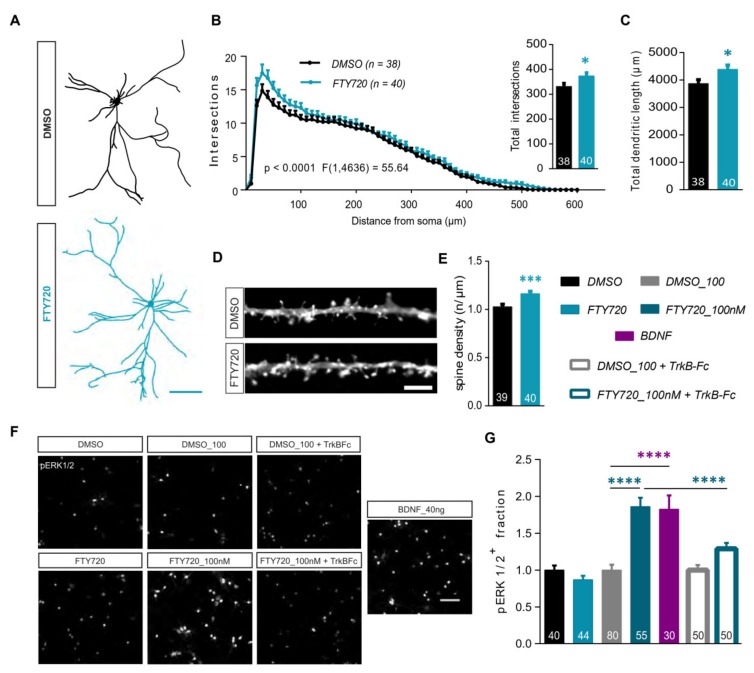
Treatment with the non-phosphorylated Fingolimod (FTY720) modulates neuronal architecture. (**A**)**|** Representative Neurolucida tracings from feGFP positive hippocampal neurons used for the Sholl analysis from cultures treated either with DMSO or 10nM FTY20 for 24 h. Scale bar: 100μm. (**B**)**|** Dendritic complexity shown by the number of dendritic intersections plotted against the distance from the soma for DMSO (black) and FTY720 (blue) treated neurons. The *F* value shows the statistical comparison between the two groups. The inset graph represents total dendritic complexity upon treatment with DMSO (black) and FTY720 (blue). (**C**)**|** Total dendritic length for DMSO (black) and FTY720 (blue) treated neurons. (**D**)**|** Representative stretches from dendrites of eGFP transfected hippocampal neurons showing dendritic spine protrusions, treated either with DMSO or FTY720 for 24h. Scale bar: 5μm. (**E**)**|** The graph shows dendritic spine density for DMSO (black) and FTY720 (blue) treated neurons**.** (**F**)**|** Representative images of fields of view (FOV) from primary hippocampal cultures stained with anti phospho-ERK1/2 antibody, 30 min post-application of one of the following: DMSO, 10nM FTY720, DMSO_100, 100nM FTY720, DMSO_100 + TrkB-Fc, 100nM FTY720 + TrkB-Fc or 40ng recombinant BDNF protein as a positive control. Scale bar: 100μm. (**G**)**|** The graph displays the fraction of pERK1/2 expressing neurons relative to the total number of MAP2^+^ neurons. The data is normalized to the respective controls and compared between the different treatment groups: DMSO (black), 10nM FTY720 (light blue solid), DMSO100 (gray), 100nM FTY720 (dark blue solid), 40ng recombinant BDNF (magenta), DMSO100 + TrkB-Fc (gray open), 100nM FTY720 + TrkB-Fc (dark blue open). All data is plotted as mean + SEM. Numbers in the bars show total number of neurons or FOV analyzed, obtained from ≥3 sets of independent experiments. Two-way ANOVA followed by Bonferroni post-hoc test was used in (**B**). For (**B**) total intersections, (**C**) and (**E**) unpaired Student’s t-test and for (**G**) one-way ANOVA with Bonferroni post-hoc was used. Denotations for significance are * *p* < 0.05, *** *p* < 0.001, **** *p* < 0.0001.

FTY720-P is the active drug form known to interact with its target S1P receptors at membrane surfaces. However, in a clinical scenario the drug is provided in the form of non-phosphorylated FTY720, due to its ability to cross the blood brain barrier and the lipid membrane. Therefore, we next wanted to investigate whether the non-phosphorylated FTY720 is capable of recapitulating the effects we observed using the phosphorylated form. Thus, mature DIV21 hippocampal cultures were treated for 24 h with 10 nM FTY720 or the relative DMSO control. First, the dendritic complexity was analyzed in feGFP expressing neurons using Sholl analysis (Figure 3A,B). The dendritic tree of FTY720 treated neurons was significantly more complex than the one of the DMSO-treated counterparts (Figure 3A,B; two-way ANOVA *F*_1,4636_ = 55.64, *p* < 0.0001). Accordingly, total dendritic complexity (Figure 3B; two-tailed unpaired *t*-test: *t* = 2.277, *df* = 76, *p* < 0.05; DMSO: 330.8 ± 13.10, FTY720: 373.4 ± 13.29) as well as total dendritic lengths (Figure 3C; two-tailed unpaired *t*-test: *t* = 2.410, *df* = 76, *p* < 0.05; DMSO: 3867 ± 142.9, FTY720: 4385 ± 159.2) showed a significant increase in the FTY720 treated group when compared to the controls. Finally, the effect of a 24h application of FTY720 was analyzed for dendritic spine density. Also here, FTY720 application led to a significant increase in dendritic spine density compared to the DMSO application (Figure 3D,E; two-tailed unpaired *t*-test: *t* = 3.550, *df* = 77, *p* < 0.001; DMSO: 1.027 ± 0.0283, FTY720: 1.162 ± 0.0256).

As shown by Deogracias et al., (2012) a 30-min FTY720-P treatment upregulated the phosphorylation of extracellular signal-regulated kinase 1/2 (pERK1/2). We tested if this ERK1/2 phosphorylation could also be enhanced by the non-phosphorylated form. Primary hippocampal neurons were treated with 10 nM FTY720 for 30 min and stained using antibodies against pERK1/2 and MAP2. A clear immunofluorescence was observed in the cytoplasm and nucleus of pERK1/2 expressing cells (Figure 3F). The number of cells positive for pERK1/2 (pERK1/2^+^) were quantified over the total of MAP2*^+^* neurons. While at a concentration of 10 nM, FTY720 did not enhance pERK1/2 expressing cells compared to its DMSO control (Figure 3F,G; one-way ANOVA *F*_6,342_ = 18.17, *p* < 0.0001; DMSO: 1.000 ± 0.0639, FTY720: 0.8692 ± 0.0558), a 10 times higher dose (100 nM), significantly increased the number of pERK1/2^+^ neurons by ~80% compared to the DMSO treated cultures (Figure 3F,G; one-way ANOVA *F*_6,342_ = 18.17, *p* < 0.0001; DMSO_100: 1.000 ± 0.07469, FTY720_100nM: 1.862 ± 0.1214). it is noteworthy that this increase in the pERK1/2^+^ fraction was comparable to the one, resulting upon a 30 min application of recombinant BDNF protein (40 ng) (Figure 3F,G; one-way ANOVA *F*_6,342_ = 18.17, *p* < 0.0001; BDNF: 1.825 ± 0.1876). We then probed whether this upregulation of pERK1/2^+^ after 100 nM FTY720 treatment could be driven by BDNF. Therefore, the 30 min treatment with 100 nM FTY720 was repeated in the presence of TrkB-Fc receptor bodies. TrkB-Fc co-application completely prevented the FTY720-induced increase in the number of pERK1/2 expressing neurons. (Figure 3F,G; one-way ANOVA *F*_6,342_ = 18.17, *p* < 0.0001; DMSO+TrkB-Fc: 1.000 ± 0.07079, FTY720_100nM+TrkB-Fc: 1.291 ± 0.07765). 

These observations confirm that the non-phosphorylated FTY720 can indeed successfully induce similar structural alterations in mature hippocampal neurons, as produced by its active phosphorylated form FTY720-P. Additionally, at a higher concentration (100 nM) FTY720 acutely upregulates phosphorylation of ERK1/2 in neurons in a BDNF-dependent fashion. 

### 2.5. Fingolimod-Phosphate (FTY720-P) Completely Rescues the Neurite Defects Observed in Young Mecp2^-/y^ Neurons and Only Partially in Cdkl5^-/y^ Neurons 

Our results so far demonstrate the ability of FTY720-P to positively modulate the dendritic architecture of hippocampal neurons in a BDNF-dependent fashion. Therefore, we wondered if the drug could be extended for use onto mouse models for neurodevelopmental diseases characterized by defects in neuronal architecture combined with BDNF deficiency. We selected the *Mecp2*^-/y^ transgenic mouse as a model for Rett Syndrome (RTT) caused by the expression of a dysfunctional Mecp2 protein. In addition, *Cdkl5*^-/y^ transgenic mice were used as a model for the cyclin-dependent kinase-like 5 (*Cdkl5*) deficiency disorder (CDD) caused by loss of function mutation in the X-linked *Cdkl5.* Both diseases are associated with abnormalities in the neuronal architecture in hippocampal and cortical neurons, including simpler dendritic arbors and reduced dendritic lengths starting already at very early stages during development [16,17,36] [21,22,37,38]. 

**Figure 4:**
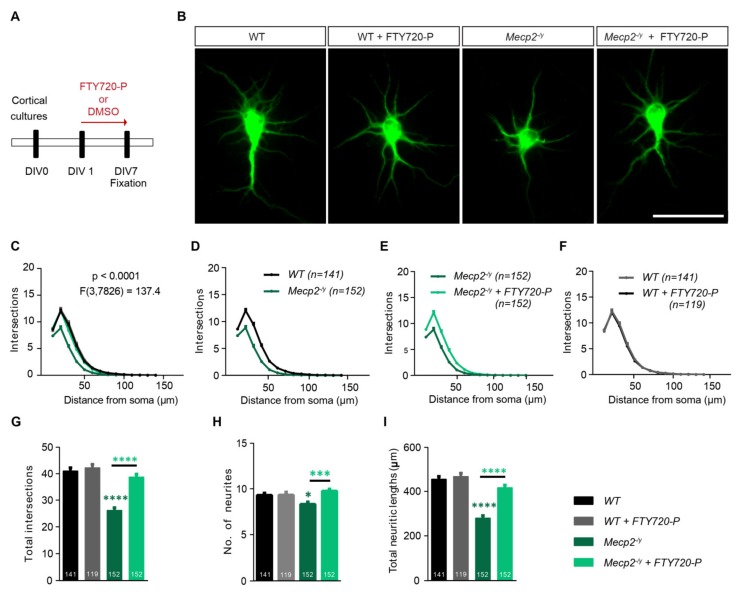
Fingolimod-phosphate (FTY720-P) completely rescues the neuronal architecture in developing *Mecp^-/y^* cortical neurons. Young DIV7 cortical cultures from *Mecp2^-/y^* mice or littermate wild type (WT) controls were allowed to develop for one week with either DMSO alone or with 10nM FTY720-P in the growth medium. (**A**) The diagram shows the scheme of treatment from culture preparation on DIV0, treatment from DIV1 to fixation on DIV7. (**B**) Micrographs showing representative fluorescent images of DIV7 WT and *Mecp2^-/y^* cortical neurons stained for MAP2 and treated with either DMSO or FTY720-P. Scale bar: 50 μm. Neurite complexity plotted as number of neurite intersections against the distance from the soma for: (**C**) all four test groups- WT (black), WT neurons treated with 10nM FTY720-P (gray), *Mecp2^-/y^* (dark green) and *Mecp2^-/y^* neurons treated with 10nM FTY720-P (light green). *F* value for comparison between all sets is described with the graph. For better visualization of differences between different pairs, Sholl curves are also plotted separately: (**D**) WT (black) and *Mecp2^-/y^* (dark green) neurons (**E**) *Mecp2^-/y^* neurons treated with 10nM FTY720-P (light green) v/s DMSO controls (dark green) and (**F**) WT neurons treated with 10nM FTY720-P (gray) v/s DMSO controls (black). (**G**) Total intersections, (**H**) number of neurites and (**I**) total length of neurites as computed for WT neurons treated with DMSO (black) or FTY720-P (gray) and *Mecp2^-/y^* treated with DMSO (dark green) or FTY720-P (light green). Data in graphs is plotted as mean + SEM. Numbers in the bars show total number of neurons analyzed for each genotype and treatment, obtained from ≥3 sets of independent experiments. Two-way ANOVA followed by Bonferroni post-hoc test was used in A. For G,H and I one-way ANOVA with Bonferroni post-hoc was used. Denotations for significance are *** *p* < 0.001, **** *p* < 0.0001.

Low-density primary cortical neurons from wild type *(*WT*)* or *Mecp2* knockout (*Mecp2^-/y^*) mice were used to test the ability of FTY720-P to rescue the impairment in the neuronal structural development typical of RTT. The DIV1 primary cortical neurons were let develop in the presence of 10 nM FTY720-P or DMSO for 7 days (Figure 4A schematic) before being fixed and stained for MAP2 (Figure 4B). Individual MAP2 positive neurons with well-defined cell bodies and neuritic protrusions were selected to analyze dendritic development using Sholl analysis (Figure 4B,C; two-way ANOVA *F*_3,7826_ = 137.4, *p* < 0.0001). WT cells treated with DMSO or FTY720-P were qualitatively indistinguishable (Figure 4B). In addition, the quantification of their neuritic complexity by Sholl analysis displayed no difference between these two treatment groups (Figure 4C,F). The *Mecp2^-/y^* neurons, on the other hand were remarkably smaller than WT with shorter and fewer neurites (Figure 4B). When compared to WT, *Mecp2^-/y^* neurons were characterized by a significantly lower neuritic complexity as shown by the Sholl analysis (Figure 4C,D). In addition, the total complexity of *Mecp2^-/y^* neurons was significantly lower compared to WT neurons (Figure 4G; one-way ANOVA *F*_3,560_ = 43.11, *p* < 0.0001; WT: 41.02 ± 1.221, *Mecp2 ^-/y^*: 26.13 ± 0.9886). While WT neurons had an average number of 9.29 ± 0.2442 primary neurites arising from the cell soma (Figure 4H; one-way ANOVA *F*_3,564_ = 6.042, *p* < 0.001), the *Mecp2^-/y^* neurons only had 8.30 ± 0.2507 (Figure 4H). However, *Mecp2^-/y^* neurons that developed for 7 days in the presence of FTY720-P showed longer and more complex neurites than the control-treated counterparts (Figure 4B). This was also true for neurite complexity which significantly increased in the FTY720-P treated group (Figure 4C,E). Moreover, compared to the WT neurons, *Mecp2 ^-/y^* neurons displayed a decrease of ~37% in total neurite intersections which was completely rescued upon treatment with FTY720-P (Figure 4G; one-way ANOVA *F*_3,560_ = 43.11, *p* < 0.0001; *Mecp2*^-/*y*^ + FTY720-P: 38.74 ± 1.098). Also, the average number of primary neurites in the *Mecp2^-/y^* cultures treated with FTY720-P was restored to WT level (Figure 4H; one-way ANOVA *F*_3,564_ = 6.042, *p* < 0.001; *Mecp2^-/y^* + FTY720-P: 9.752 ± 0.2274). Lastly, the neurites of *Mecp2^-/y^* neurons were overall significantly shorter than the WT neurons (Figure 4I) and treatment with FTY720-P completely rescued their total neurite length (Figure 4I; one-way ANOVA *F*_3,560_ = 49.24, *p* < 0.0001; WT: 455.7 ± 12.93, *Mecp2^-/y^*: 279.6 ± 10.69, *Mecp2^-/y^* + FTY720-P: 417.2 ± 11.79). The FTY720-P treatment in WT cultures did not alter total neurite intersections (Figure 4G; one-way ANOVA *F*_3,560_ = 43.11, *p* < 0.0001; WT: 41.02 ± 1.221, WT+FTY720-P: 42.25 ± 1.307) and length (Figure 4I; one-way ANOVA *F*_3,560_ = 49.24, *p* < 0.0001; WT: 455.7 ± 12.93, WT+FTY720-P: 467.8 ± 14.95). 

**Figure 5:**
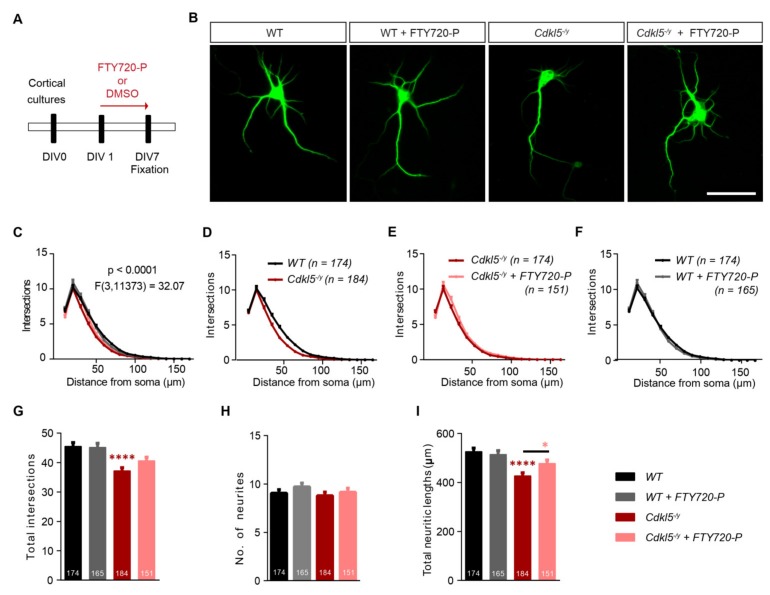
Fingolimod-phosphate (FTY720-P) shows mild rescue in neuronal morphology of young cortical neurons from *Cdkl5^-/y^* mice. Young DIV7 cortical cultures from mice carrying a *Cdkl5^-/y^* mutation or littermate wild type (WT) controls, were grown in presence of DMSO alone or 10 nM FTY720-P in the growth medium. (**A**) The diagram shows the scheme of treatment from culture preparation on DIV0, treatment from DIV1 to fixation on DIV7. (**B**) Micrographs display representative DIV7 MAP2 positive cortical neurons. The WT and *Cdkl5^-/y^* neurons were both treated either with DMSO or FTY720-P. Scale bar: 50 μm. (**C**) Neurite complexity for both genotypes treated with DMSO and FTY720-P was analyzed and plotted as number of neurite intersections against the distance from the soma. *F* value for comparison between the four sets is described in the graph. The Sholl curve for following genotype and treatment sets are also displayed separately (**D**) WT (black) v/s *Cdkl5^-/y^* (dark red), (**E**) *Cdkl5^-/y^* neurons treated with 10nM FTY720-P (rosa) v/s DMSO controls (dark red), (**F**) WT neurons treated with 10nM FTY720-P (gray) v/s DMSO controls (black). (**G**) Total intersections, (**H**) number of neurites and (**I**) total length of neurites as computed for WT neurons treated with DMSO (black) or FTY720-P (gray) and *Cdkl5^-/y^* neurons treated with DMSO (dark red) or FTY720-P (rosa). Data in graphs is represented as mean + SEM. Numbers in the bars show total number of neurons analyzed for each genotype and treatment, obtained from ≥3 sets of independent experiments. Two-way ANOVA followed by Bonferroni post-hoc test was used in (**A**). For (**G**,**H**) and (**I**) one-way ANOVA with Bonferroni post-hoc was used. Denotations for significance are * *p* < 0.05, **** *p* < 0.0001.

Next, DIV 1 WT or *Cdkl5^-/y^* cortical cultures were cultured in the presence of 10 nM FTY720-P or DMSO until DIV 7 (Figure 5A). MAP2 positive neurons from each treatment group show cell body with several neurites of different lengths (Figure 5B). WT neurons treated with DMSO alone or with FTY720-P displayed no difference in overall appearance (Figure 5B) and the quantification of their neuritic complexity showed no significant difference both for the Sholl analysis (Figure 5C,F) and their total complexity (Figure 5G; one-way ANOVA *F*_3,669_ = 10.48, *p* < 0.0001; WT: 45.51 ± 1.306, WT+FTY720-P: 45.17 ± 1.393). On the other hand, neurons lacking *Cdkl5* exhibited significant structural deficits identifiable by their less complex neurite morphology and shorter neurites (Figure 5B). The Sholl analysis revealed significantly lower complexity for *Cdkl5^-/y^* neurites in comparison to WT neurons (Figure 5C,D; two-way ANOVA *F*_3,11373_ = 32.07, *p* < 0.0001). FTY720-P in the growth medium resulted in a mild, albeit significant increase in the neurite complexity of *Cdkl5^-/y^* neurons (Figure 5C,E). While total neuritic complexity of FTY720-P treated *Cdkl5^-/y^* neurons was only slightly higher than control *Cdkl5^-/y^* neurons (Figure 5G; one-way ANOVA *F*_3,669_ = 10.48, *p* < 0.0001; *Cdkl5^-/y^*: 37.18 ± 1.104, *Cdkl5^-/y^* + FTY720-P: 40.63 ± 1.204), the total neuritic length was significantly enhanced (Figure 5I; one-way ANOVA *F*_3,670_ = 10.48, *p* < 0.0001; *Cdkl5^-/y^*: 427.2 ± 12.53, *Cdkl5^-/y^* + FTY720-P: 478.1 ± 13.89). Although the average number of primary neurites in *Cdkl5^-/y^* neurons was slightly lower than the WT, it was statistically not significant (Figure 5H; one-way ANOVA *F*_3,669_ = 1.162, *p* = 0.3235; WT: 9.086 ± 0.3673, *Cdkl5^-/y^*: 8.804 ± 0.3707, *Cdkl5^-/y^* + FTY720-P: 9.166 ± 0.4110). WT neurons treated with FTY720-P showed a mild, though not statistically significant increase in the number of primary neurites (Figure 5H; one-way ANOVA *F*_3,669_ = 1.162, *p* = 0.3235; WT: 9.086 ± 0.3673, WT+FTY720-P: 9.780 ± 0.3909) and did not show any changes in their total neurite length (Figure 5I; one-way ANOVA *F*_3,670_ = 10.48, *p* < 0.0001; WT: 526.1 ± 14.77, WT±FTY720-P: 514.7 ± 15.48).

In conclusion, these results illustrate that the deficiency of Mecp2 and Cdkl5 protein respectively leads to severely stunted growth of immature developing cortical neurons as seen by reduction in neurite length and complexity. These defects can be entirely rescued by FTY720-P application into the growth medium for *Mecp2^-/y^* neurons and only partially for *Cdkl5^-/y^* neurons.

## 3. Discussion

Our results describe a role of the immunomodulatory drug Fingolimod in modulating the dendritic architecture of healthy mature primary hippocampal neurons. Indeed, Fingolimod application to hippocampal primary neurons results in a significant increase both in dendritic complexity and in dendritic spine density, associated to a higher proportion of mature, mushroom-like spines. Moreover, we show that a treatment with Fingolimod is able to prevent the structural defects described in hippocampal neurons from the mouse models of two neurodevelopmental disorders, namely Rett syndrome and Cdkl5 deficiency disorder.

### 3.1. Fingolimod Regulates Neuronal Architecture and the Expression of Activity-Related Genes in Healthy Mature Neurons in a BDNF-Dependent Manner

Fingolimod crosses the blood brain barrier and accumulates within the brain where, binding to S1PRs exerts a series of direct functions on CNS cells including enhanced neuroprotective effects, reduced inflammation and improvement of neuronal pathology in different neurological disorders. Moreover, Fingolimod rescues the cognitive impairment and associated dendritic spine pathology observed in mouse models of Alzheimer and Huntington disease [12,13,14] indicating that, at least in part, the beneficial effects of Fingolimod may derive from its ability to modulate neuronal architecture. However, very little is currently known about the possible effects of Fingolimod in modulating the dendritic and spine architecture of healthy neurons. We show here that Fingolimod application to primary hippocampal neurons results in increased spine density, associated with a higher proportion of mushroom-like, mature spines identified by their positivity for SynapsinI/II. While application of Fingolimod has been previously shown to promote neurite outgrowth in developing neurons [25], we show that this effect also extends to mature healthy hippocampal neurons, showing an increase in dendritic complexity after treatment. While these observations in vitro do not allow us to conclude that a similar effect could be observed in vivo, they do indicate a role for Fingolimod in modulating dendritic and spine architecture to promote neuronal connectivity within primary hippocampal cultures and are supported by previous reports showing an increase in network activity after Fingolimod application [11]. 

Several lines of evidence indicate that Fingolimod promotes BDNF synthesis and secretion both in vivo and in vitro [11,12,13,15]. Moreover, BDNF has been shown to modulate dendritic and spine architecture in an age-dependent manner [3,33]. Here, we show that co-application of the BDNF scavenging TrkB receptor bodies completely prevents the increase in dendritic complexity, spine density and maturation observed upon Fingolimod-P treatment in hippocampal primary neurons indicating the requirement for BDNF secretion in this context. Indeed, several in vitro studies support a role for BDNF signaling in modulating the dendritic architecture during development [3,33,39] and in regulating dendritic spine density and morphology in mature hippocampal neurons [31,32]. We report similar observations on dendritic complexity, spine density and spine morphology of mature hippocampal neurons upon Fingolimod application. It is noteworthy, that although the effects observed upon treatment in mature, healthy neurons are relatively mild they are significant and consistent in their manner of expression. Indeed, Fingolimod specifically increases the complexity of the proximal dendritic tree. A specific effect on proximal dendrites has been previously described in cortical neurons overexpressing BDNF [29] and after exogenous BDNF treatment in cultured hippocampal neurons [30]. However, while in our study Fingolimod affects mature neurons already after 24 h of treatment, the effects of exogenous BDNF application required days [30]. While a clear effect of the exogenous application of BDNF in modulating dendritic spine density in hippocampal neurons could be observed in some studies [31,32], our laboratory failed to reproduce it [33], possibly due to differences in culture conditions. However, in the current study we observe a significant, BDNF-dependent increase in dendritic spine density upon Fingolimod treatment. This observation suggests a higher efficacy of the Fingolimod-induced release of endogenous BDNF compared to its exogenous application, possibly due to a requirement for a specific action at the release site rather that a global effect, as per bath application. On the other hand, it should be noted that previous studies did not observe a regulation of BDNF synthesis upon Fingolimod treatment in vivo [11] indicating the need for further studies.

The effects of BDNF on dendritic spine density have been show to occur in an activity-dependent manner [33] and the positive modulation of dendrite architecture by BDNF occurs via the activity-dependent expression of the cAMP response element-binding protein (CREB) [30]. Interestingly, an increase in the phosphorylation of CREB and of network activity was observed in Fingolimod treated cortical cultures [11] suggesting that the structural changes upon its application, could be mediated via an increase in neuronal activity and CREB phosphorylation. This increased activity is likely since we show a significantly higher number of c-Fos expressing neurons upon Fingolimod application. The immediate early gene *c-fos* is known to be upregulated upon membrane depolarization via CREB phosphorylation and is a commonly used marker for recently activated neurons [40]. Such a sustained increase in c-Fos positive neurons, as seen 24 h after the beginning of Fingolimod treatment could support a positive BDNF-feedback loop and thus contribute to the structural alterations. Indeed, *c-fos* is known to participate in a positive feedback loop downstream of TrkB signaling promoting BDNF expression [41]. Finally, our data show also in hippocampal neurons an increase in the phosphorylation of the extracellular signal-regulated kinase 1/2 (ERK1/2) in treated cultures as previously reported for cortical neurons [11]. The increase in the proportion of pERK1/2^+^ cells in Fingolimod treated cultures was comparable to the one observed in BDNF treated ones and was, as for c-Fos expression reduced to control levels upon co-treatment with TrkB-Fc receptor bodies again supporting the necessity of BDNF secretion for the Fingolimod induced effects in hippocampal neurons. 

### 3.2. Fingolimod Treatment Rescues the Impaired Neurite Outgrowth in Cortical Neurons in Two X-linked Neurodevelopmental Disorders

While a growing body of evidence supports the benefits of modulating BDNF-TrkB signaling in several neurological conditions, its inability to cross the blood brain barrier as well as the need for temporally and spatially regulated BDNF signaling limit its direct use in therapy. Alternative methods for modulating BDNF levels within specific brain areas include drug-induced BDNF expression. Among these drugs Fingolimod has been successfully used to rescue the cognitive impairment observed in many neurological diseases. However, still, little is known about the cellular mechanisms underlying its beneficial action. Our study adds to this notion, by analyzing the cellular effects of Fingolimod in two X-linked neurodevelopmental disorders, namely Rett syndrome (RTT) and Cdkl5 deficiency disorder (CDD). Both disorders show severe anomalies in neuronal structures both in patients [42] and in the relative mouse models: *Mecp2* [17] or *Cdkl5* knockout mice [37]. Our results confirmed the altered neuronal phenotype in developing cortical neurons derived from *Mecp2^-/y^* as well as *Cdkl5^-/y^* mice. The knockout neurons were found to be significantly simpler in complexity and shorter when compared to their relative controls. Fingolimod application completely rescued the impairment in neurite growth in *Mecp2*^-/y^ cortical neurons. While our results cannot exclude a BDNF-independent activity of Fingolimod, it is interesting that the onset of RTT-like phenotypes in *Mecp2* knockouts is associated with the absence of the typical increase in BDNF levels observed postnatally. The *bdnf*/*Mecp2* double knockout shows exacerbated symptoms and an earlier disease onset [18], indicating a link between the impaired BDNF expression and the RTT pathology. Accordingly, promoting BDNF/TrkB signaling via Fingolimod injection significantly improved motor behavior and the average lifespan of *Mecp2* mutant mice [11]. The current results support the hypothesis that Fingolimod might rescue neuronal structure in *Mecp2*^-/y^ mice, possibly in a BDNF-dependent manner. It is important to note that our findings indicate benefits of Fingolimod by rescuing the typical defects in neuronal architecture at an early developmental stage. However, whether a rescue of the morphological phenotype could be obtained in fully developed neurons in juvenile or adult knockout animals needs further investigation. While application of a TrkB agonist has been shown to rescue both functional and structural impairments in the perirhinal cortex of *Cdkl5*^-/y^ mice [23], little is known about the role of BDNF in the pathology of CDD in contrast to RTT. Our results show that application of Fingolimod results in a partial rescue of the structural impairments observed in *Cdkl5*^-/y^ cortical neurons. While our current results cannot exclude an effect of Fingolimod independent of its ability to promote BDNF release, the partial rescue in *Cdkl5^-/y^* could be explained by looking at the position of BDNF along the Mecp2 and Cdkl5 downstream signaling pathways. Indeed, while Mecp2 functions upstream of BDNF to regulate its activity-dependent transcription [43], Cdkl5 lies downstream of BDNF-TrkB signaling to activate Rac1 [44]. Thus, while modifying endogenous BDNF levels using Fingolimod may compensate for its reduced levels in *Mecp2*^-/y^ neurons, it may not rescue the effects of the loss of the intermediate signaling protein in *Cdkl5*^-/y^ neurons. The partial rescue observed possibly indicates an indirect, neuroprotective action of Fingolimod in this context. 

In summary, the results of our study can be divided in two main independent parts. On one side, our study reports specific BDNF-dependent effects of the immunomodulatory drug Fingolimod in modulating the dendrite architecture of mature, healthy CNS neurons. In addition, our observations add to the understanding regarding the cellular mechanisms underlying the positive effects of a Fingolimod treatment in two highly relevant X-linked neurodevelopmental disorders.

## 4. Materials and Methods

### 4.1. Animals

All studies including wild-type (WT) mice were done using C57Bl/6J mice bred in the mouse facility of the TU Braunschweig. The *Mecp2* null mouse strain, originally purchased from Jackson Laboratories (003890 B6.129P2(C)-Mecp2<tm1.1Bird>/J), was backcrossed and maintained on a clean CD1 background [45]. The *Cdkl5* null mouse strain was originally kindly donated by Dr Elisabetta Ciani (University of Bologna). C57BL/6 *Cdkl5* heterozygous females were crossed with CD1 WT male mice for at least ten generations. Timed-pregnant females were generated by overnight crossing wild type or hemizygous CD1 males with *Mecp2*^+/−^ or *Cdkl5^+/-^* CD1 females. The *Mecp2* and the *Cdkl5* null mutant strains were bred in the laboratory of Prof. Nicoletta Landsberger where the experiments with these mice were performed (University of Milan, Milan). Genotypes were assessed by PCR on genomic DNA purified from tails to distinguish knockouts and littermate WT controls. Mice were housed on a 12 h light/dark cycle in a temperature-controlled environment (21 ± 2 °C) with food and water provided ad libitum. All procedures concerning animals were performed in accordance with the European Union Communities Council Directive (2010/63/EU) and were approved by the animal welfare representative of the TU Braunschweig and the LAVES (Oldenburg, Germany, Az. §4 (02.05) TSchB TU BS) as well as by the Italian Council on Animal Care, the Italian Government decree No. 210/2017 and the San Raffaele Scientific Institutional Animal Care and Use Committee in accordance with the Italian law. 

### 4.2. Reagents and Stock Solutions

Fingolimod hydrochloride: FTY720 (USBiological, Swampscott, MA, USA) and Fingolimod-Phosphate: FTY720-P (Cayman chemical, Ann Arbor, MI, USA), were dissolved in Dimethyl sulfoxide (DMSO, anhydrous; ThermoFisher Scientific,Waltham, MA, USA). FTY720-P required gentle sonication for 2–5 min for dissolution. Stock solution for FTY720 (100mM), FTY720-P (1mM) and control DMSO vials were stored at −20 °C until used. Recombinant Human BDNF protein (R&D systems, Minneapolis, MN, USA) was dissolved in sterile PBS with 0.1% BSA at a concentration of 50 ng/μL. BDNF scavenging TrkB-Fc receptor bodies (Recombinant Human TrkB-Fc Chimera, R&D systems, Minneapolis, MN, USA) were reconstituted in sterile PBS at 50 μg/mL. BDNF and TrkB-Fc were stored at −70 °C until used.

### 4.3. Primary Hippocampal Cultures

Primary mouse hippocampal and cortical cultures were prepared on embryonic day 18. The pregnant female was rapidly killed by cervical dislocation, the abdomen was sterilized, and the embryos were collected under sterile conditions. Post-decapitation the embryonic brains were quickly isolated and immersed in ice-cold Gey’s balanced salt solution supplemented with glucose and with a pH adjusted to 7.3. The hippocampi and/or cortices were dissected, incubated in Trypsin-EDTA (Sigma-Aldrich, St. Louis, MO, USA) at 37 °C for 30 min and further dissociated mechanically using a Pasteur pipette. The cells were re-suspended in Gibco Neurobasal medium ( ThermoFisher Scientific, Waltham, MA, USA) supplemented with 2% B27(Invitrogen, Carlsbad, CA, USA) (*v/v*), 10% N2 and 0.5 mM Gibco glutamax ( ThermoFisher Scientific, Waltham, MA, USA) and plated at high (7 × 10^4^/cm^2^) or low density (3.5 × 10^4^/cm^2^) on 12 mm glass coverslips previously coated with poly-L-lysine (Sigma-Aldrich, St. Louis, MO, USA). The cultures were incubated at 37 °C, 5% CO_2_ and 95% O_2_ until usage. Medium change was done once a week by replacing 20% of the medium. The C57Bl/6J WT hippocampal cultures were fixed at DIV21 whereas *Mecp2* and *Cdkl5* cortical cultures, at DIV7. 

### 4.4. Transfection and Treatments

At DIV20, mature hippocampal neurons were transfected using Lipofectamine2000 (ThermoFisher Scientific, Waltham, MA, USA) as per manufacturer’s instructions. 0.8μg farnesylated-eGFP (Clonetech: pEGFP-F, Mountain View, CA, USA) expressing plasmid were used for each well. Then, 24-h after transfection, the cells were treated with 2, 10, or 100 nM Fingolimod phosphate (FTY720-P) or 10 and 100 nM Fingolimod (FTY720) and the respective DMSO controls diluted into Neurobasal medium without supplements. All FTY720-P concentrations used in the study were in the nM range. Since the physiological levels of Fingolimod crossing the blood brain barrier and accumulating in local neuronal vicinity cannot be clearly estimated, our dosage selection was based on a previous report by Deogracias et al. (2012). They showed nM ranges of FTY720-P to maximally enhance BDNF levels (2-fold) in cortical cultures. This effect however was reduced when the drug concentration reached μM range. In some experimental sets DMSO and FTY720-P (2 nM) treated cultures were co-treated with TrkB-Fc receptor bodies (500 ng/mL). For pERK1/2 experiments, BDNF (40 ng/mL) treatment was used as a positive control. The cells were fixed 24 h or, for pERK 30 min after treatment. *Mecp2* and *Cdkl5* knockout cortical cultures were treated starting at DIV1 by adding 100 μL of fresh Neurobasal medium with supplements containing either 10nM FTY720-P or DMSO to the wells and were fixed at DIV7. 

### 4.5. Immunocytochemistry

The cultured cells were fixed for 10 min in ice cold-Paraformaldehyde (4% in PBS) and washed 3 times with PBS. Permeabilization and blocking of unspecific binding sites was done at room temperature (RT) for 1 h with PBS containing 0.2% Triton X-100 (Sigma-Aldrich, St. Louis, MO, USA) and 1.5% Normal Goat Serum (NGS, ThermoFisher Scientific, Waltham, MA, USA). Next, the cells were incubated overnight with primary antibodies diluted in 1.5% NGS + 0.2% Triton X-100 containing PBS at 4 °C on a rocker. The following primary antibodies were used: mouse anti-MAP2 (1:1000, Sigma-Aldrich, St. Louis, MO, USA)), rabbit anti-cFos (1:10,000, Synaptic systems, Goettingen, NI, Germany), rabbit anti-Phospho-MAPK/Erk1/2 (1:1000, Cell signaling technology, Danvers, MA, USA), rabbit anti- SynapsinI/II (1:1000, Millipore, Burlington, MA, USA). Secondary antibodies were diluted 1:500 in PBS in varying combinations: anti-rabbit IgG, anti-mouse IgG conjugated with appropriate cyanine fluorophores Cy2/Cy3 or Cy5 (Jackson ImmunoResearch Labs, West Grove, PA, USA). The secondary antibody incubation was done for 2h at RT on a rocker. Finally, the cultures were counterstained with 4′,6-diamidino-2-phenylindole (DAPI) diluted 1:1000 in PBS for 10 min. The coverslips were then washed with PBS and mounted onto glass slides using anti fading Fluoro-Gel embedding medium (Electron Microscopy Sciences, Hatfield, PA, USA).

### 4.6. Image Acquisition and Analysis

Fluorescence microscopy was performed using a Zeiss Axioplan2 microscope equipped with an ApoTome module (Carl Zeiss AG, Oberkochen, BW, Germany), 10× (NA 0.3), 20× (NA 0.8) and 63× (oil, NA 1.4) objectives along with a Zeiss AxioCam MRm camera. For Sholl analysis [46], single feGFP transfected neurons were imaged using a 10× objective. A healthy neuron was identified by a well-defined cell body and absence of irregular membranous protrusions around the soma. Dentate gyrus cells, readily identifiable via their smaller cell body and thicker, shorter dendrites were excluded from the analysis. The neuronal images were subsequently imported into the Neurolucida 6 software (MicroBrightField, Williston, VT, USA), the dendritic trees were traced and thereafter their complexity was analyzed using Neurolucida explorer by quantifying the number of intersecting dendrites at every 10 μm incremental step, starting at the cell body. The total dendritic complexity, as the sum of all dendritic intersections and total dendritic length were also calculated for each cell. Spatially isolated and non-overlapping secondary dendrites were selected for spine density analysis and classification. These were imaged using a 63× (oil, NA 1.4) objective and z-sectioned at 0.5 μm increments using the ApoTome module for structured illumination. Dendritic spine density was quantified by dividing the number of dendritic spines counted with ImageJ (NIH, Bethesda, MD, USA) per the length in µm of the analyzed dendritic segment. Dendritic spines were classified as previously described [24] based on their length (from spine base at dendrite to spine head tip: Spine_len_), head width (Head_max_) and neck width (Neck_min_). These factors were measured manually for individual spines using ImageJ and the spines were sorted into different subtypes based on the following criteria:

$${\mathrm{Spine}}_{\mathrm{len}}\;\leq \;1\mu m\;\&\;{\mathrm{Head}}_{\max}/{\mathrm{Neck}}_{\min}\;\leq \;1\;\mu m:\;\mathrm{Stubby}$$

$${\mathrm{Spine}}_{\mathrm{len}}>\;1\mu m\;\&\;{\mathrm{Head}}_{\max}/{\mathrm{Neck}}_{\min}\;<1.5\;\mu m:\;\mathrm{Thin}$$

$${\mathrm{Spine}}_{\mathrm{len}}\;\geq \;3\mu m\;\&\;{\mathrm{Head}}_{\max}/{\mathrm{Neck}}_{\min}\;\leq \;1\;\mu m:\;\mathrm{Filopodia}$$

$${\mathrm{Head}}_{\max}/{\mathrm{Neck}}_{\min}\;\geq \;1.5\;\mu m:\;\mathrm{Mushroom}$$

In some experimental sets, additional immunolabeling for SynapsinI/II was performed. The proportion of the co-localization between immunoreactive SynapsinI/II positive puncta and feGFP-labelled dendritic spines was analyzed using ImageJ (NIH). A feGFP- labelled spine (green) was considered co-localized with SynapsinI/II puncta (red), when a resulting yellow punctum was observed within the same focal plane or two planes above and below. Furthermore, quantification of c-Fos (Cy3) and pERK (Cy3) immunopositivity was done over MAP2+ (Cy2) neurons: neurons were imaged with 10× (NA 0.3) objective under different fluorescent channels. In DIV7, low density cultures, isolated cells were identified based on their MAP2 staining to avoid cells with overlapping neurites. With 20× (NA 0.8) objective, neurons were imaged and analyzed for neurite morphology using Sholl analysis. The analysis was always done by an experimenter blinded for the different genotypes and treatments.

### 4.7. Data Representation and Statistics

Each treatment was performed in duplicate or triplicate for each experimental set and data from at least three sets of independent experiments were grouped together in Microsoft Excel (Microsoft Corporation, Redmond,WA, USA). For plotting the graphs and performing the appropriate statistical tests, the data sets were imported into Prism 6 (GraphPad Software Inc, San Diego, CA, USA ). Unless otherwise mentioned, all data in the graphs are represented as mean +SEM. For comparing only two treatment groups a two-tailed unpaired Student’s *t*-test was used. The comparison of more than two experimental groups was done by using a one-way ANOVA followed by a Bonferroni’s multiple comparisons post-hoc test. For Sholl analysis measurements, a regular two-way ANOVA was performed followed by a Bonferroni’s multiple comparisons post-hoc test. Significance was considered for *p* value < 0.05.
